# A genetic variant in the *LDLR* promoter is responsible for part of the LDL-cholesterol variability in primary hypercholesterolemia

**DOI:** 10.1186/1755-8794-7-17

**Published:** 2014-04-07

**Authors:** Isabel De Castro-Orós, Javier Pérez-López, Rocio Mateo-Gallego, Soraya Rebollar, Marta Ledesma, Montserrat León, Montserrat Cofán, Jose A Casasnovas, Emilio Ros, Jose C Rodríguez-Rey, Fernando Civeira, Miguel Pocoví

**Affiliations:** 1 Departamento de Bioquímica y Biología Molecular y Celular, Universidad de Zaragoza, C. Pedro Cerbuna 12, 50009 Zaragoza, Spain; 2 Unidad de Lípidos y Laboratorio de Investigación Molecular, Hospital Universitario Miguel Servet, Instituto Aragonés de Ciencias de la Salud (IACS), Zaragoza, Spain; 3 Departamento de Biología Molecular. Facultad de Medicina, Universidad de Cantabria and Instituto de Formación e Investigación Marques de Valdecilla (IFIMAV), Santander, Spain; 4 Unidad de Investigación Cardiovascular, Instituto Aragonés de Ciencias de la Salud (IACS), Zaragoza, Spain; 5 Aragon Workers Health Study, Zaragoza, Spain; 6 Servei d’Endocrinologia i Nutrició, Institut d’Investigacions Biomèdiques August Pi Sunyer (IDIBAPS), Hospital Clínic, Barcelona and Ciber Fisiopatología de la Obesidad y Nutrición (CIBERobn), Instituto de Salud Carlos III (ISCIII), Barcelona, Spain

**Keywords:** LDL-cholesterol, Hypercholesterolemia, Variant, Gene regulation, Polygenic, LDLR

## Abstract

**Background:**

GWAS have consistently revealed that *LDLR locus* variability influences LDL-cholesterol in general population. Severe *LDLR* mutations are responsible for familial hypercholesterolemia (FH). However, most primary hypercholesterolemias are polygenic diseases. Although *Cis*-regulatory regions might be the cause of LDL-cholesterol variability; an extensive analysis of the *LDLR* distal promoter has not yet been performed. We hypothesized that genetic variants in this region are responsible for the *LDLR* association with LDL-cholesterol found in GWAS.

**Methods:**

Four-hundred seventy-seven unrelated subjects with polygenic hypercholesterolemia (PH) and without causative FH-mutations and 525 normolipemic subjects were selected. A 3103 pb from *LDLR* (-625 to +2468) was sequenced in 125 subjects with PH. All subjects were genotyped for 4 SNPs (rs17242346, rs17242739, rs17248720 and rs17249120) predicted to be potentially involved in transcription regulation by *in silico* analysis. EMSA and luciferase assays were carried out for the rs17248720 variant. Multivariable linear regression analysis using LDL-cholesterol levels as the dependent variable were done in order to find out the variables that were independently associated with LDL-cholesterol.

**Results:**

The sequencing of the 125 PH subjects did not show variants with minor allele frequency ≥ 10%. The T-allele from g.3131C > T (rs17248720) had frequencies of 9% (PH) and 16.4% (normolipemic), p < 0.00001. Studies of this variant with EMSA and luciferase assays showed a higher affinity for transcription factors and an increase of 2.5 times in *LDLR* transcriptional activity (T-allele vs C-allele). At multivariate analysis, this polymorphism with the lipoprotein(a) and age explained ≈ 10% of LDL-cholesterol variability.

**Conclusion:**

Our results suggest that the T-allele at the g.3131 T > C SNP is associated with LDL-cholesterol levels, and explains part of the LDL-cholesterol variability. As a plausible cause, the T-allele produces an increase in *LDLR* transcriptional activity and lower LDL-cholesterol levels.

## Background

Hypercholesterolemia due to high plasma levels of low-density lipoprotein cholesterol (LDL-C) is the main risk factor for cardiovascular diseases. The individual concentration of LDL-C depends on the interplay between genetic and environmental factors. Severe primary hypercholesterolemias are often monogenic disorders, such as familial hypercholesterolemia (FH) associated with mutations in coding regions of *LDLR*, *APOB*, or *PCSK9* genes [1-4]. However, no functional defects are detected in these genes in most subjects with primary hypercholesterolemia, albeit, in comparison with FH, a milder phenotype and lower family penetrance are usually present. Actually, most of these subjects receive a diagnosis of polygenic hypercholesterolemia (PH), and it is believed that common genetic variants in the population are responsible for this phenotype [5]. It has been recently published that a substantial number of subjects with FH without a known mutation have increased the number of polymorphisms associated with higher LDL-C levels, therefore they might have a polygenic cause [6,7].

Several Genome Wide Association Studies (GWAS) carried out during the past decade have revealed that variability at the *LDLR locus* is associated with LDL-C levels [8-11]. These GWAS clearly signal the *LDLR* as a candidate gene for PH. As an example, the minor allele of the rs6511720 polymorphism in *LDLR* was associated with a reduction of 6.99 mg/dl in total cholesterol levels [12] and this variant has been included in the LDL-C gene score proposed by Talmud, however its functionality is still unknown [6].

*Cis*-regulatory sequences control when, where, and with what intensity genes are expressed [13]. Genetic variation in these regions that influence gene expression are widespread in the human genome and are responsible for most of the inter-individual variability of normal phenotypes, but also for complex and polygenic diseases [14-18]. Various rare mutations in the promoter region of *LDLR* have been characterized in subjects with FH [19-22]. However, whether or not common genetic variants in the regulatory region of *LDLR* are associated with non-FH primary hypercholesterolemia had not been studied previously and is the hypothesis of the present study.

## Methods

### Study subjects

The PH group was composed of 477 unrelated subjects recruited at two lipid clinics and a large car factory involved in a prospective study of cardiovascular health named Aragon Workers’ Health Study (AWHS). The three centers are located in north-eastern Spain, and the population groups involved share the same genetic background. Participants were selected according to the following criteria, absence of prior cardiovascular disease, LDL-C ≥ 90th percentile of a Spanish reference population [23], triglyceride levels ≤ 200 mg/dl, BMI < 30 kg/m^2^, and no mutations in the *LDLR*, *APOB* or *PCSK9* genes. Secondary causes of hypercholesterolemia were excluded in all subjects by clinical history and blood tests. A normolipemic group of 525 unrelated healthy individuals was selected from the AWHS with LDL-C ≤ 90th percentile and TG ≤ 200 mg/dl. Exclusion criteria for controls were the same as those for PH cases, except that they were not tested for FH-causing mutations and had not received prior treatment with hypolipidemic drugs. All subjects provided written consent to a protocol approved by the ethical review boards of the two participating hospitals and the Comité Ético de Investigación Clínica de Aragón, CEICA, for AWHS subjects.

### Clinical and laboratory characteristics

All subjects were assessed for family history of early-onset coronary heart disease, clinical history, medication use, demographic characteristics, adiposity measures, and cardiovascular risk factors. Fasting blood for biochemical profiles was drawn after ≥ 4 weeks without hypolipidemic drug treatment.

Subjects were categorized as never smokers and current or former smokers. Hypertension was defined as systolic Blood Pressure (BP) ≥ 140 mm Hg, diastolic BP ≥ 90 mm Hg, or current use of antihypertensive medication. Diabetes was defined as a fasting glucose level ≥ 126 mg/dL on at least two occasions or treatment with anti-diabetic agents.

Blood glucose, High-density lipoprotein cholesterol (HDL-C), cholesterol and triglyceride levels were determined by standard methods. LDL-C was estimated as total cholesterol – HDL-C–TG/5. Apolipoprotein (apo) B and lipoprotein(a) were determined in the lipid clinics by using immunoturbidimetry (Unimate 3, Roche, Basel, Switzerland) and by the nephelometry analyzer IMMAGE 800 (Beckman Coulter, USA) in AWHS subjects.

### Genetic analyses

DNA was isolated from EDTA blood samples following standard procedures. A molecular diagnosis of FH was ruled out in all hypercholesterolemic subjects from the lipid clinics by using the LIPOchip® genetic diagnostic platform [24]. The LIPOchip® platform (v3 to v9.1) contains the most frequent Spanish mutations and CNVs in *LDLR*, *APOB* and 4 in *PCSK9*. According to the LIPOchip® protocol, the coding sequences, the intron-exon boundaries as well as the promoter of the *LDLR* and part of the *APOB* exon 26 were sequenced in those individuals with negative results.

The variability of the regulatory region of the *LDLR* gene was studied in 125 PH subjects. To this end, a 3.103 Kb fragment of the *LDLR* gene [ENSG:00000130164], including the promoter (from -625 to +2478, considering the A nucleotide from ATG as +1) was sequenced (Additional file 1: Table S1).

In order to predict possible transcription changes due to the presence of a variant, known SNPs located in the 3 Kb fragment upstream of the transcription initiation site were analyzed with both MatInspector and CHIP Mapper [25]. These *in silico* analyses reported four SNPs, namely rs17242346, rs17242739, rs17248720 and rs17249120, potentially involved in the *LDLR* regulation. We genotyped these polymorphisms, located in the *LDLR* 5’ promoter, by iPlex technology based on a MassARRAY® platform (Sequenom®, Valencia, Spain) in all the PH (n = 477) and control (n = 525) subjects. The reproducibility of this method was confirmed by obtaining consistent genotypes in 16 replicated samples.

### Electrophoretic mobility shift assays

Preparation of nuclear extracts from sub-confluent HepG2 cell cultures and Electrophoretic Mobility Shift Assays (EMSAs) were done as reported by Riancho et al. [26]. The gel bands were analyzed in an ODYSSEY infrared imaging system (Li-Cor Biosciences, Lincoln, NE). The relative affinity of each allele was analyzed by competing it with increasing amounts of the unlabelled allele that had shown stronger binding. The bands were quantified and the affinities were calculated by representing the inverse of band intensity versus the excess of unlabelled oligonucleotide. A higher slope indicates lower oligonucleotide-protein affinity. The experiment was performed in triplicate to confirm results.

### Plasmid construct

A fragment of 2457 bp of the *LDLR* promoter from c.-2,335 to c.67 + 36 was amplified by Polymerase Chain Reaction (PCR) from the DNA of individuals carrying the g.3131C > T (rs17248720) variants in homozygosis, g.3131C and g.3131 T alleles. The oligonucleotides introduce a SacI and NheI restriction site on the 5’ and 3’ end of the amplified fragment, respectively. The PCR product was digested with SacI and NheI and cloned into the digested luciferase reporter gene vector pGL3-Basic (Promega, Madison, WI). The identity of the *LDLR* promoter within the cloned DNA fragment, the C or T variant allele, and the absence of other polymorphisms or mutations were verified by sequencing of the resulting plasmid.

### Cell culture and transient transfection

HepG2 cells were cultured in Dulbecco’s Modified Eagle’s Medium (DMEM) supplemented with 10% fetal bovine serum (FBS), 100 U/ml penicillin and 100 μg/ml streptomycin and transfected with a jetPEI™-Hepatocyte. As a positive control and for normalization pGL3-Basic and pRL-TK plasmids (Promega, Madison, WI) were used. In the first experiment we added either g.3131C_LDLR-pGL3 or g.3131T_LDLR-pGL3. Cells were processed and Luciferase assays performed as previously described in De Castro-Orós et al. [21].

### Statistical analyses

Data are presented as means ± Standard Deviation (SD) for continuous variables (medians and interquartile ranges for variables with a skewed distribution) and as frequencies or percentages for categorical variables. Differences in mean values were assessed by using unpaired *t-*tests or the Mann–Whitney *U-*test, as appropriate. Categorical variables were compared using χ^2^ test. Genotype prevalence was estimated as observed proportions. First, all subjects were distributed into quintiles according to the LDL-C levels; these groups were composed of 199 individuals. Second, the quintile distribution was made in control subjects and in each group 105 subjects were included. Quintiles distribution of T-carriers and T-allele frequency for rs17248720 SNP in the *LDLR* gene were calculated in control subjects according to LDL-C levels, p-value was calculated by Chi-square test corrected by Bonferroni. Bivariate logistic regression analysis was used to determine the odds ratios (OR) and 95% confidence intervals (CI) of *LDLR* genotypes in cases versus controls. Regression coefficients (β) and multivariate logistic regression with the forward selection based on likelihood ratios was used to examine the effect of *LDLR* genotypes on LDL-C. Variables shown in Table 1 showing P values < 0.20 in the bivariate analysis were introduced in the model. All analyses were performed with the Statistical Package for the Social Sciences software, version 16.0 (SPSS Inc., Chicago, IL), with significance set at *P <* 0.05.

**Table 1:** Characteristics of the hypercholesterolemic and normolipemic groups

	**Polygenic hypercholesterolemia (n=477)**	**Normolipemics (n=525)**	P value*
**Men (%)**	57.4	90.9	< 0.001
**Age (years)**	51 (41-56)	51 (42-54)	0.004
**BMI (kg/m**^**2**^**)**	26 (24-28)	27 (25-29)	< 0.001
**Waist circumference (cm)**	92 (84-98)	95 (88-101)	< 0.001
**Smoking status (%)**			0.019
**Active smokers**	24.8	37.3	
**Non-smokers**	49.9	37.0	
**Past smokers**	25.3	25.7	
**Hypertension (%)**	20.7	20.2	0.885
**Laboratory variables (mg/dL)**			
**Fasting glucose**	91 (84-98)	94 (87-102)	< 0.001
**Total cholesterol**	290 ± 42	202 ± 32	< 0.001
**Triglycerides**	108 (83-137)	100 (72-136)	0.019
**HDL-cholesterol**	53 (46-65)	53 (47-61)	0.026
**LDL-cholesterol**	207 (186-231)	127 (107-147)	< 0.001
**Lipoprotein(a)**	29 (13-63)	19 (8-51)	< 0.001
**Apolipoprotein B**	148 ± 27	97 ± 22	< 0.001

Data shown for quantitative variable when normal distribution are means ± SD or median and interquartile range for variables with non-normal distribution. Data shown for qualitative variable are percentages. *Calculated by unpaired *t*-test or Mann–Whitney U–test, as appropriate.

## Results

Table 1 shows the clinical and biochemical characteristics of study subjects. By study design, PH subjects disclosed higher levels of total cholesterol, LDL-C, and apo B than controls, while triglycerides were similar between groups. The sequencing of 3.103 Kb of the relevant *LDLR* promoter region was carried out in 125 PH subjects and only six (rs12981050, rs17242353, rs57217136, rs59281581, rs60173709 and rs6511720) appeared with frequencies for the minor allele between 0.012 and 0.085 (Additional file 2: Table S2).

We also analyzed four polymorphisms revealed by prediction software [25,27] to be located in potential regulatory elements in the distal promoter. The minor allele frequencies of these variants are shown in Table 2. All the analyzed polymorphisms were compatible with Hardy-Weinberg expectations. The analyzed SNPs rs17242346, rs17242739 and rs17249120 presented a minor allele frequency below 10% and only the minor allele of the rs17242346 was more frequent in the control group. One of the four variants, rs17248720, showed highly significant (p < 0.00001) allelic distribution differences between the PH and normolipemic populations. In fact, when the frequency was calculated separately within the three hypercholesterolemic populations, all showed minor allele frequencies significantly lower than those in the control group.

**Table 2:** Polymorphisms analyzed in PH and normolipemic populations by iPlex technology

	**Polygenic hypercholesterolemia**				**Controls**	
**Polymorphisms**	**Zaragoza N=252**	**Barcelona N=127**	**AWHS N=98**	**Total N=477**	**AWHS N=525**	**P value**
**rs17242346 (g.3724G>A)**	0.107	0.071	0.122	0.100	0.080	0.113
**rs17242739 (g.3627C>A)**	0.000	0.008	0.000	0.000	0.000	0.280
**rs17248720 (g.3131C>T)**	0.090	0.083	0.102	0.090	0.160	< 0.0001
**rs17249120 (g.4440G>A)**	0.004	0.000	0.020	0.003	0.010	0.082

AWHS, Aragon Workers’ Health Study. Zaragoza, Barcelona and AWHS were the three hypercholesterolemic populations analyzed. Variables were compared using χ^2^ test. Genotype prevalences were estimated as observed proportions.

When control groups were distributed in quintiles (Q) according to LDL-C levels the Q2 showed more T-allele carriers than the Q3, Q4 and Q5 (0.371 > 0.352 > 0.267 > 0.200) (p < 0.05) (Table 3). When all study subjects were distributed in quintiles we observed that this frequency was higher in Q3 than Q4 and Q5 with an almost significant p-value (Additional file 3: Table S3).

**Table 3:** LDL cholesterol quintile distribution of frequencies of genotypes CT and TT and T-allele frequency for rs17248720 polymorphism in LDLR gene in Control subjects

	**Q1**	**Q2**	**Q3**	**Q4**	**Q5**	**p**
**LDL-C**	86 (77.5-95.0)	112 (107-116)	127 (124-131)	143 (139-147)	163 (155-168)	
**(mg/dl)**						
**CT+TT genotype**	0.324	0.371	0.352	0.267	0.200	0.046
**T-allele frequency**	0.176	0.195	0.195	0.143	0.109	

LDL-C, LDL cholesterol measured by median and interquartiles. p: p-value was calculated by Chi-square test and corrected by Bonferroni.

Table 4 shows the results of multivariable linear regression analysis using LDL-C levels as the dependent variable. The variables that were independently associated with LDL-C were lipoprotein(a) concentration, *LDLR* g.3131C > T, and age, in this order, explaining together 10% of its variability in men. The results were similar when all study subjects were considered and did not substantially differ when triglycerides, HDL-C, fasting glucose, and waist circumference were forced into the model. The age- and gender-adjusted OR of disclosing PH for subjects bearing the *LDLR* g.3131 T allele was 0.478 (IC 0.342-0.667, p < 0.001).

**Table 4:** Variables independently associated with LDL-cholesterol concentration by multivariate lineal regression analysis*

**Variables**	**Standardized coefficient (β)**	**P**	**Adjusted R**^**2**^
Men			
Lipoprotein(a), mg/dL	0.190	< 0.001	0.039
*LDLR* g.3131C > T, yes/no	-0.179	< 0.001	0.070
Age, years	0.177	< 0.001	0.100
All study subjects			
Age, years	0.236	< 0.001	0.054
Lipoprotein(a), mg/dL	0.172	< 0.001	0.083
*LDLR* g.3131C > T, yes/no	-0.146	< 0.001	0.103

*Variables allowed to enter the model were those with P values < 0.20 in the bivariate analysis.

**Figure 1:**
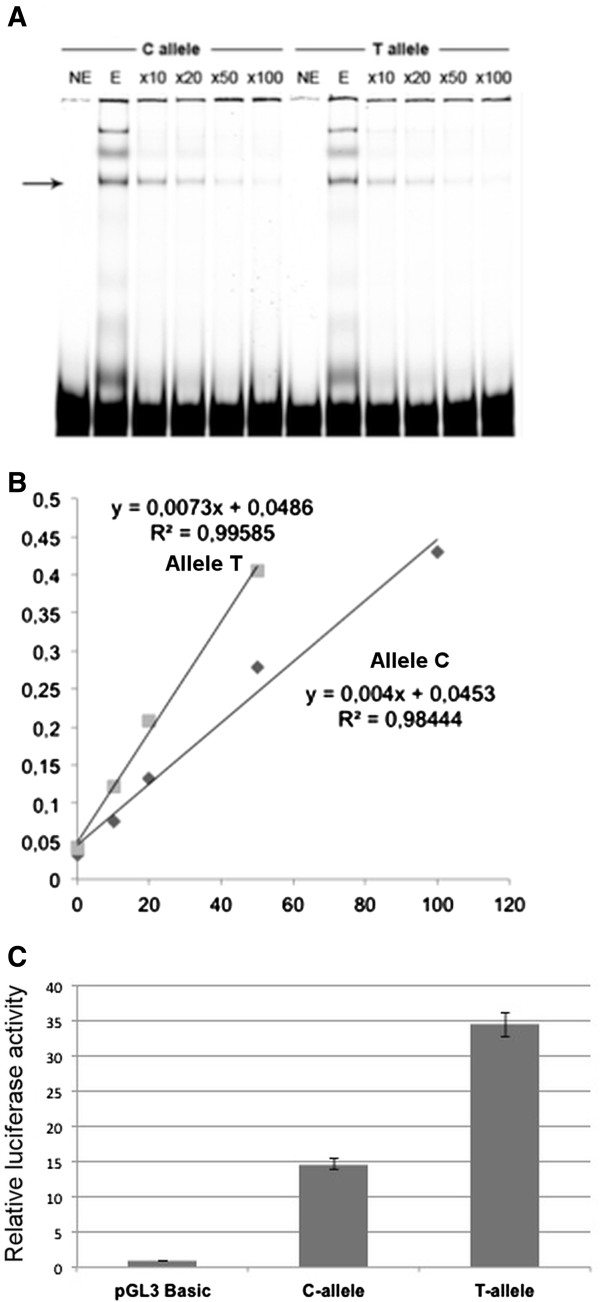
**Functional assays for g.3131C > T SNP (rs17248720). A)** EMSA assay carried out with probes containing the C or T allele for the g.3131 SNP (rs17248720) in the *LDLR* gene. NE, no HepG2 extract added. E, nuclear extract from HepG2 was added, ×10, ×20, ×50 and ×100 mean 10, 20, 50 and 100 times excess of unlabelled C-allele oligonucleotide. **B)** The inverse of band densities from the EMSA shown in Figure 1A were plotted against the excess of unlabelled allele C oligonucleotide. **C)** Influence of g.3131C > T (rs17248720) variant on *LDLR* promoter strength in transient transfection assay in FBS supplemented media. Fragments containing, none, either C or T allele were cloned in the promoterless plasmid pGL3-Basic and transfected to FBS supplemented media cultures of HepG2. Measurements of luciferase activity were assayed in cell extracts 36 h after transfection. The results were expressed as the media of three experiments carried out in triplicate considering pGL3-Basic expression as value 1.

To find out whether the C to T change alters the ability of the polymorphism region to bind transcription factors, we performed an EMSA using oligonucleotides specific for each variant. The results (Figure 1A) showed noticeable differences in affinity for nuclear proteins between the two alleles. After the binding was competed with increasing amounts of an unlabelled oligonucleotide corresponding to allele C, the inverse of the intensity of the major band was plotted against the excess of unlabelled oligonucleotide. The result (Figure 1B) indicated that the T allele was easily displaced from the protein-DNA complex (slope 0.0073 vs 0.004).

In order to test if the different affinity between variants influences the transcription of the *LDLR* gene, two constructs carrying both variants of SNP g.3131C > T were transfected in HepG2 cells. In comparison with cells transfected with the g.3131C allele, those having the g.3131 T construct showed a significant increase of 2.5 times (p < 0.01) of transcriptional activity (Figure 1C).

## Discussion

In this study we used the novel approach of searching for common polymorphisms located in the 5’UTR region of the *LDLR* that might be involved in the pathogenesis of primary hypercholesterolemia because of their possible influence in the transcription of the *LDLR* gene. The major conclusions drawn from this study are that, the *LDLR* g.3131C > T polymorphism in the 5’ region of the *LDLR* is responsible for a major effect on LDL-C concentration because of the increase in the transcriptional activity of the *LDLR* gene, and also indicates that the *LDLR* variation is associated with PH.

It has been shown that 58.9% of the variants involved in regulation changes are located within the first 500 bp upstream of the transcription start site, but 12.8% are more than 1 kb upstream and show transcription factor switching, with each allele having a higher affinity for a different transcription factor [15,28]. As the *LDLR* promoter is well-conserved in most of its positions among chimps and humans, the sequencing of a 3103 pb fragment, from -625 to +2478 of the ENSG00000130164 reference, showed only 6 variants, all located within the first and second introns of the *LDLR* gene and with ≤ 8.5% minor allele frequency.

Simultaneously to the sequencing of the *LDLR* promoter we analyzed four polymorphisms that appeared to be relevant in the regulation of *LDLR* expression according to the bioinformatics analysis. We found that only the variant g.3131C > T had a different distribution of genotypes between a control group of 525 individuals carefully selected among a working population of 1137 subjects and a group of 477 hypercholesterolemic individuals without known FH-causing mutations (p < 0.00001). A lower frequency of the minor T allele of this SNP was observed in the total hypercholesterolemic population and also in each of the three subgroups taken separately. Surprisingly, the T-allele is also the ancestral one, as it has been observed by sequence comparison between 20 eutherian mammals, and, according to our results, this allele would have a protective effect against hypercholesterolemia.

The g.3131C > T is placed in a large linkage disequilibrium (LD) block. In this work we had sequenced this LD region in 125 subjects, including the rs6511720 SNP, and we have calculated the LD between these SNPs (r^2^ = 0.211 and D’ = 0.487) not observing an association in this group.

A limitation to the study would be the diagnosis with the LIPOchip® in the PH subjects as non-carriers of FH causal mutations. It would be remotely possible to have missed a variant located in a region not sequenced with this platform, although coefficients for specificity and sensitivity are 99.7% and 99.9%, respectively [29]. Another limitation to the study is the different gender distribution in the PH and control groups. However, regression analyses in men and in the overall population showed a similar independent inverse association of the *LDLR* g.3131C > T variant with LDL-C that, together with the lipoprotein(a) concentration and age, explained ≈ 10% of LDL-C variability. The increase of the ancestral (and minor) allele frequency in populations with a lower concentration of LDL-C comparing to PH group and in replication cohorts suggests that T-allele of the g.3131C > T variant in the *LDLR* gene protects against hypercholesterolemia. This observation might be explained by the lower affinity of the g.3131 T variant for some repressors of *LDLR* gene transcription, and as a consequence, it would increase the transcriptional activity of the LDLR gene comparing with the wild-type allele (Figure 1).

Taking together the genetic analyses and the functional assays, our results suggest that the ancestral and minor allele T at g.3131C > T is associated with LDL-C levels and explains part of the LDL-C variability. This variant is located in a regulatory element, and the shift of nucleotide C to T produce a change in the affinity for transcription factors as well as an increase of 2.5 times in the transcriptional activity of the *LDLR* gene explaining the protective effect of the T allele. Further studies aimed to expand knowledge on the regulatory elements in the distal region of the *LDLR* promoter are warranted. Future studies should also consider the effect of this variant on the LDL-C response to lipid-lowering drugs or lifestyle changes.

The association of the rs17248720 polymorphism with hypercholesterolemia confirms previous observations from GWAS suggesting that non-coding variants located at the *LDLR* locus are associated with blood lipid levels, including LDL-C [8,9,12,13]. While studies attempt to find new loci implicated in the hereditability of lipid levels, the strongest locus associated with LDL-C continues to be that containing the *LDLR* gene [10].

## Conclusions

We have identified a variant located in the distal promoter of the *LDLR* gene, g.3131C > T (rs17248720) that would explain part of the variability o the LDL cholesterol levels. The ancestral T-allele at this site plays a protective role against hypercholesterolemia by changing the affinity of this region for transcription factors leading to an increase of 2.5 times in the transcriptional activity of the *LDLR* gene.

## Abbreviations

Apo: Apolipoprotein; AWHS: Aragon Workers Health Study; BMI: Body mass index; BP: Blood pressure; EMSA: Electrophoretic mobility shift assay; FH: Familial hypercholesterolemia; GWAS: Genome wide association studies; HDL-C: HDL cholesterol; LD: Linkage disequilibrium; LDL-C: LDL cholesterol; PH: Polygenic hypercholesterolemia; SNPs: Single nucleotide polymorphisms; TG: Triglycerides.

## Competing interests

The authors declare that they have no competing interest.

## Authors’ contributions

IDCO carried out the genetic studies, the sequence alignment, participated in functional studies and statistical analysis and drafted the manuscript. JPL and SR developed the *in silico* analysis and the functional studies. RMG, ML, ML, MC and JAC participated in the acquisition of data and samples. ER, JCRR, FC and MP conceived of the study and participated in its design and coordination. All authors have been involved in revising critically for important intellectual content and have given final approval of the version to be published.

## Pre-publication history

The pre-publication history for this paper can be accessed here:

http://www.biomedcentral.com/1755-8794/7/17/prepub

## Supplementary Material

Additional file 1: Table S1 Primers designed for sequencing a 3.103 Kb fragment from the -625 to +2478 position in the *LDLDR* gene. Click here for file

Additional file 2: Table S2 Frequencies of variants in the regulatory region of *LDLR* found in 125 PH subjects by sequencing. Click here for file

Additional file 3: Table S3 LDL cholesterol quintile distribution of frequencies of genotypes CT and TT and T-allele frequency for rs17248720 polymorphism in LDLR gene in all study subjects. Click here for file
