# Supplementary material for: A genetic variant in the LDLR promoter is responsible for part of the LDL-cholesterol variability in primary hypercholesterolemia
Source: BMC Med Genomics. 2014 Apr 7;7:17. doi: 10.1186/1755-8794-7-17 (PMC4021749; doi:10.1186/1755-8794-7-17)
Supplement: Additional file 1: Table S1 — Primers designed for sequencing a 3.103 Kb fragment from the -625 to +2478 position in the LDLDR gene. [file 1755-8794-7-17-S1.docx]

**Additional Table 1.** Primers designed for sequencing a 3.103 Kb fragment from the -625 to +2478 position in the *LDLDR* gene.

| **Name** | **Sequence** | **Name** | **Sequence** |  |
| --- | --- | --- | --- | --- |
| LDLR Prom 01 F | 5’ TGATTGATCAGTGTCTAT 3’ | LDLR Prom 01 R | 5’ TTTTAACCCGTGAAGCTC 3’ |  |
| LDLR Prom 02 F | 5’ GGTGAAGACATTTGAAAA 3’ | LDLR Prom 02 R | 5’ CCTCCAGCCGTTTGGGAA 3’ |  |
| LDLR Prom 03 F | 5’ TGTCCCCCAAGTCTCCACA 3’ | LDLR Prom 03 R | 5’ GTGGGAGTGATCCCTTGT 3’ |  |
| LDLR Prom 04 F | 5’ GCTCAGGGGTCCCGATCC 3’ | LDLR Prom 04 R | 5’ TCCGGGGACCCGGGACTTGT 3’ | |
| LDLR Prom 05 F | 5’ GTTTCCGCTGTGCTCTGT 3’ | LDLR Prom 05 R | 5’ GAGGCTGTCTCTCTGCAA 3’ |  |
| LDLR Prom 06 F | 5’ TAGCCTAGAAAAGGATTG 3’ | LDLR Prom 06 R | 5’ CTTTTGTAAAACTGAGTG 3’ |  |
| LDLR Prom 07 F | 5’ GAGAAAATGTTAAGGAAG 3’ | LDLR Prom 07 R | 5’ CCGGAAATGACAAGGGAA 3’ |  |
