# Supplementary material for: A genetic variant in the LDLR promoter is responsible for part of the LDL-cholesterol variability in primary hypercholesterolemia
Source: BMC Med Genomics. 2014 Apr 7;7:17. doi: 10.1186/1755-8794-7-17 (PMC4021749; doi:10.1186/1755-8794-7-17)
Supplement: Additional file 2: Table S2 — Frequencies of variants in the regulatory region of LDLR found in 125 PH subjects by sequencing. [file 1755-8794-7-17-S2.docx]

**Additional Table 2.** Frequencies of variants in the regulatory region of *LDLR* found in 125 PH subjects by sequencing.

| **Polymorphism** | **Location** |  | **Minor allele frequency** |
| --- | --- | --- | --- |
| **rs12981050** | c.67+121C>T | Intron 1 | 0.074 |
| **rs17242353** | c.67+515C>T | Intron 1 | 0.048 |
| **rs57217136** | c.67+833T>C | Intron 1 | 0.085 |
| **rs59281581** | c.67+838_67+839insG | Intron 1 | 0.012 |
| **rs60173709** | c.67+1698delT | Intron 1 | 0.080 |
| **rs6511720** | c.67+2015G>T | Intron 1 | 0.075 |
