# Supplementary material for: A genetic variant in the LDLR promoter is responsible for part of the LDL-cholesterol variability in primary hypercholesterolemia
Source: BMC Med Genomics. 2014 Apr 7;7:17. doi: 10.1186/1755-8794-7-17 (PMC4021749; doi:10.1186/1755-8794-7-17)
Supplement: Additional file 3: Table S3 — LDL cholesterol quintile distribution of frequencies of genotypes CT and TT and T-allele frequency for rs17248720 polymorphism in LDLR gene in all study subjects. [file 1755-8794-7-17-S3.docx]

**Additional Table 3.** LDL cholesterol quintile distribution of frequencies of genotypes CT and TT and T-allele frequency for rs17248720 polymorphism in LDLR gene in all study subjects.

|  | **Q1** | **Q2** | **Q3** | **Q4** | **Q5** | **p-value** |
| --- | --- | --- | --- | --- | --- | --- |
| **LDL-C (mg/dl)** | 99 (86-173) | 120 (112-189) | 133 (126-206) | 151 (142-225) | 172 (162-254) |  |
| **CT + TT genotype** | 0.236 | 0.286 | 0.352 | 0.276 | 0.201 | 0.069 |
| **T-allele frequency** | 0.118 | 0.143 | 0.176 | 0.138 | 0.100 |  |

LDL-C: LDL cholesterol measured by median and interquartiles. P-value was calculated by Chi-square test and corrected by Bonferroni.
